# Association of thiamine administration and prognosis in critically ill patients with heart failure

**DOI:** 10.3389/fphar.2023.1162797

**Published:** 2023-03-23

**Authors:** Rui Yang, Jiasheng Huang, Yumei Zhao, Jia Wang, Dongdong Niu, Enlin Ye, Suru Yue, Xuefei Hou, Lili Cui, Jiayuan Wu

**Affiliations:** ^1^ Clinical Research Service Center, Affiliated Hospital of Guangdong Medical University, Zhanjiang, China; ^2^ Guangdong Engineering Research Center of Collaborative Innovation Technology of Clinical Medical Big Data Cloud Service in Medical Consortium of West Guangdong Province, Affiliated Hospital of Guangdong Medical University, Zhanjiang, China; ^3^ Guangdong Key Laboratory of Age-Related Cardiac and Cerebral Diseases, Institute of Neurology, Affiliated Hospital of Guangdong Medical University, Zhanjiang, China

**Keywords:** thiamine, heart failure, in-hospital mortality, intensive care unit, MIMIC-Ⅳ database

## Abstract

**Background:** Thiamine deficiency is common in patients with heart failure, and thiamine supplement can benefit these patients. However, the association between thiamine administration and prognosis among critically ill patients with heart failure remains unclear. Thus, this study aims to prove the survival benefit of thiamine use in critically ill patients with heart failure.

**Methods:** A retrospective cohort analysis was performed on the basis of the Medical Information Mart of Intensive Care-Ⅳ database. Critically ill patients with heart failure were divided into the thiamine and non-thiamine groups depending on whether they had received thiamine therapy or not during hospitalization. The association between thiamine supplement and in-hospital mortality was assessed by using the Kaplan−Meier (KM) method and Cox proportional hazard models. A 1:1 nearest propensity-score matching (PSM) and propensity score-based inverse probability of treatment weighting (IPW) were also performed to ensure the robustness of the findings.

**Results:** A total of 7,021 patients were included in this study, with 685 and 6,336 in the thiamine and non-thiamine groups, respectively. The kaplan−meier survival curves indicated that the thiamine group had a lower in-hospital mortality than the none-thiamine group. After adjusting for various confounders, the Cox regression models showed significant beneficial effects of thiamine administration on in-hospital mortality among critically ill patients with heart failure with a hazard ratio of 0.78 (95% confidence interval: 0.67–0.89) in the fully adjusted model. propensity-score matching and probability of treatment weighting analyses also achieved consistent results.

**Conclusion:** Thiamine supplement is associated with a decreased risk of in-hospital mortality in critically ill patients with heart failure who are admitted to the ICU. Further multicenter and well-designed randomized controlled trials with large sample sizes are necessary to validate this finding.

## Introduction

Heart failure is a syndrome caused by heart dysfunction, and it is the end stage of all heart diseases. Recently, the Global Burden of Disease 2019 study indicates that approximately 56 million people worldwide live with heart failure, making it a major threat to human health and social development (Wei et al., 2022). In the United States, approximately 10%–51% of patients hospitalized with heart failure require ICU treatment, and in-hospital mortality for patients with heart failure who are admitted to the ICU is 10.6%, which is higher than that for all patients with heart failure (4.0%) (Safavi et al., 2013; Peng et al., 2022). Although a variety of classical drug therapy for heart failure has been widely used, including angiotensin-converting enzyme inhibitors (ACEIs), angiotensin receptor blockers (ARBs), beta-blockers, and aldosterone receptor antagonists, the mortality rate remains high. Therefore, new therapeutic interventions are urgently necessary to improve patient outcomes.

An imbalance in energy production and expenditure is associated with heart failure (Murashige et al., 2020). Micronutrient deficiency is common in patients with heart failure and it may cause poor clinical outcomes in these patients because micronutrient deficiency could reduce energy production in the myocardium (Lennie et al., 2018). Thiamine, a water-soluble vitamin, consists of a methylene bridge between a pyrimidine ring and a thiazole ring, and it is essential for the functioning of multicellular organisms. Thiamine has three forms, namely, thiamine monophosphate, thiamine pyrophosphate (TPP), and thiamine triphosphate (Kerns and Gutierrez, 2017). TPP is the main form of thiamine utilization in the human body. Under the action of thiamine pyrophosphokinase, thiamine entering cells is converted into TPP, which becomes a coenzyme of α-ketoglutarate dehydrogenase, pyruvate dehydrogenase, transketolase, and branched α-ketoate dehydrogenase complex, which is involved in cell energy metabolism (Abdou and Hazell, 2015; Polegato et al., 2019). Thiamine deficiency can decrease the production of nicotinamide adenine dinucleotide phosphate and adenosine triphosphate, which reduced the activity of transketolase and weakened the transketolase action of the pentose phosphate pathway, thereby affecting organs sensitive to hypoxia, such as the brain and heart, resulting in impaired utilization of glucose (Calderón-Ospina and Nava-Mesa, 2020). In addition, thiamine deficiency can lead to neurotransmitter changes, oxidative stress response, lactic acidosis, inflammation, apoptosis, and blood−brain barrier dysfunction (Smith et al., 2021).

These important effects of thiamine indicate the importance of thiamine supplementation in critically ill patients. For example, thiamine combined with hydrocortisone and ascorbic acid to form HAT therapy in patients with sepsis is associated with improved organ dysfunction, reduced sequential organ failure assessment scores, increased lactate clearance, and decreased mortality (Marik et al., 2017; Litwak et al., 2019). Moreover, the application of thiamine can significantly reduce the mortality of patients with ventilator-associated pneumonia and acute kidney injury in the ICU settings (Li et al., 2022; Zhang et al., 2022). Thiamine deficiency, resulting in the accumulation of pyruvate and its conversion to lactic acid, causes a decrease in peripheral resistance, thereby increasing venous return to the heart (preload). This increased preload combined with myocardial dysfunction has been proposed as the etiological basis of congestive heart failure in thiamine deficiency (Ahmed et al., 2015). Some studies have shown that thiamine supplementation can improve cardiac function in patients with heart failure, but little consistent evidence has been found on whether thiamine use can improve survival outcomes in these patients (Schoenenberger et al., 2012; Smithline et al., 2019). Compared with general hospitalized patients with heart failure, critically ill patients with heart failure likely develop thiamine deficiency because of malnutrition, increased metabolic status, and diuretic use. Therefore, considerable attention should be paid to thiamine supplementation in critically ill patients with heart failure. This study aimed to evaluate the association between thiamine administration and in-hospital mortality in critically ill patients with heart failure based on the Medical Information Mart for Intensive Care (MIMIC)-IV database, which will provide a reference for guiding rational drug use to improve prognoses.

## Materials and methods

### Data source

Data of this study were extracted from the MIMIC-IV database (https://mimic-iv.mit.edu/). The MIMIC-IV, an update to the MIMIC-III, is a large, single-center, and freely available medical information database maintained by Beth Israel Deaconess Medical Center. This database contains more than 250,000 emergency department admissions and more than 60,000 ICU stays from 2008 to 2019. The patient information in this database was anonymous; thus, informed consent need not to be obtained. The authors have completed the corresponding training courses and obtained the certificates (No: 10012145) to gain access to the database.

### Participants

Critically ill patients diagnosed with heart failure according to the International Classification of Disease 9 and 10 codes were initially screened. The inclusion criteria were as follows: 1) age ≥18 years old and 2) first admission to ICU. Patients were excluded in accordance with the exclusion criteria: 1) Stay in ICU for less than 2 days, 2) more than 15% of personal data missing, and 3) had diseases that weren’t suitable for thiamine therapy, such as stone diseases.

### Data extraction

The structured Query Language with PostgreSQL (version 9.6) was applied to extract data on the first day of admission from MIMIC-Ⅳ (Yang et al., 2020; Wu et al., 2021). The following variables were selected: 1) Demographic characteristics, including age, gender, ethnicity, and body mass index; 2) comorbidities, including myocardial infarct, hypertension, diabetes, liver disease, chronic renal disease, peripheral vascular disease, cerebrovascular disease, chronic pulmonary disease, malignant cancer, and sepsis; 3) scoring systems, including the Charlson comorbidity index, Glasgow Coma Scale, Sequential Organ Failure Assessment, and Acute Physiology Score Ⅲ; 4) vital signs, including heart rate, respiratory rate, systolic blood pressure, diastolic blood pressure, temperature, partial pressure of oxygen (PO_2_), partial pressure of carbon dioxide (PCO_2_), oxygen saturation (SpO_2_), and urine output; 5) laboratory tests, including hematocrit, hemoglobin, platelets, white blood cell, anion gap, bicarbonate, blood urea nitrogen (BUN), calcium, chloride, creatinine, glucose, sodium, potassium, hydrogen ion concentration (pH), lactate, prothrombin time, and N-terminal pro-brain natriuretic peptide (NT-proBNP); and 6) clinical therapy, including ACEIs, ARBs, implantable cardioverter defibrillator, beta-blockers, diuretics, renal replacement therapy, mechanical ventilation, and vasopressor. The primary outcome of this study was in-hospital mortality, which is defined as all-cause mortality during hospitalization. In order to minimize the bias caused by missing data, variables with over 30% missing data were removed from the analysis dataset, and others were duplicated using multiple imputation (Cummings, 2013). As a popular approach for addressing the presence of missing data, multiple imputation is a two-stage approach where missing values are imputed a number of times using a statistical model based on the available data and then inference is combined across the completed datasets.

### Statistical analysis

Based on whether thiamine was used or not, patients were divided into two groups, namely, the non-thiamine group and thiamine group. Continuous variables were presented as medians with interquartile ranges (IQRs), and categorical variables were expressed as the number of cases and percentages (%). The between-group difference was compared by using the Wilcoxon rank-sum test for continuous variables and the chi-square test for categorical variables. The Kaplan-Meier (KM) method was applied to draw survival curves for in-hospital mortality and a log-rank test was conducted to determine the difference between the two groups. Cox proportional hazard models with hazard ratios (HRs) and 95% confidence intervals (CIs) were used to assess the effect of thiamine use on prognosis by adjusting various confounders, including demographic features, comorbidities, scoring systems, vital signs, laboratory tests, and clinical therapy (Liu et al., 2022). Potential multicollinearity among variables was tested using the variance inflation factor (VIF), with a VIF ≥5, indicating the presence of multicollinearity. A 1:1 nearest propensity-score matching (PSM) and propensity-score-based inverse probability of treatment weighting (IPW) were performed to ensure the robustness of the findings (Austin and Stuart, 2017). Subgroup analyses were performed to assess the effect of thiamine use on in-hospital mortality, including demographic features and comorbidities. Two-sided *p* values less than 0.05 were considered statistically significant. All statistical analyses were conducted using R (version 4.2.1).

## Results

### Baseline feature

**FIGURE 1:**
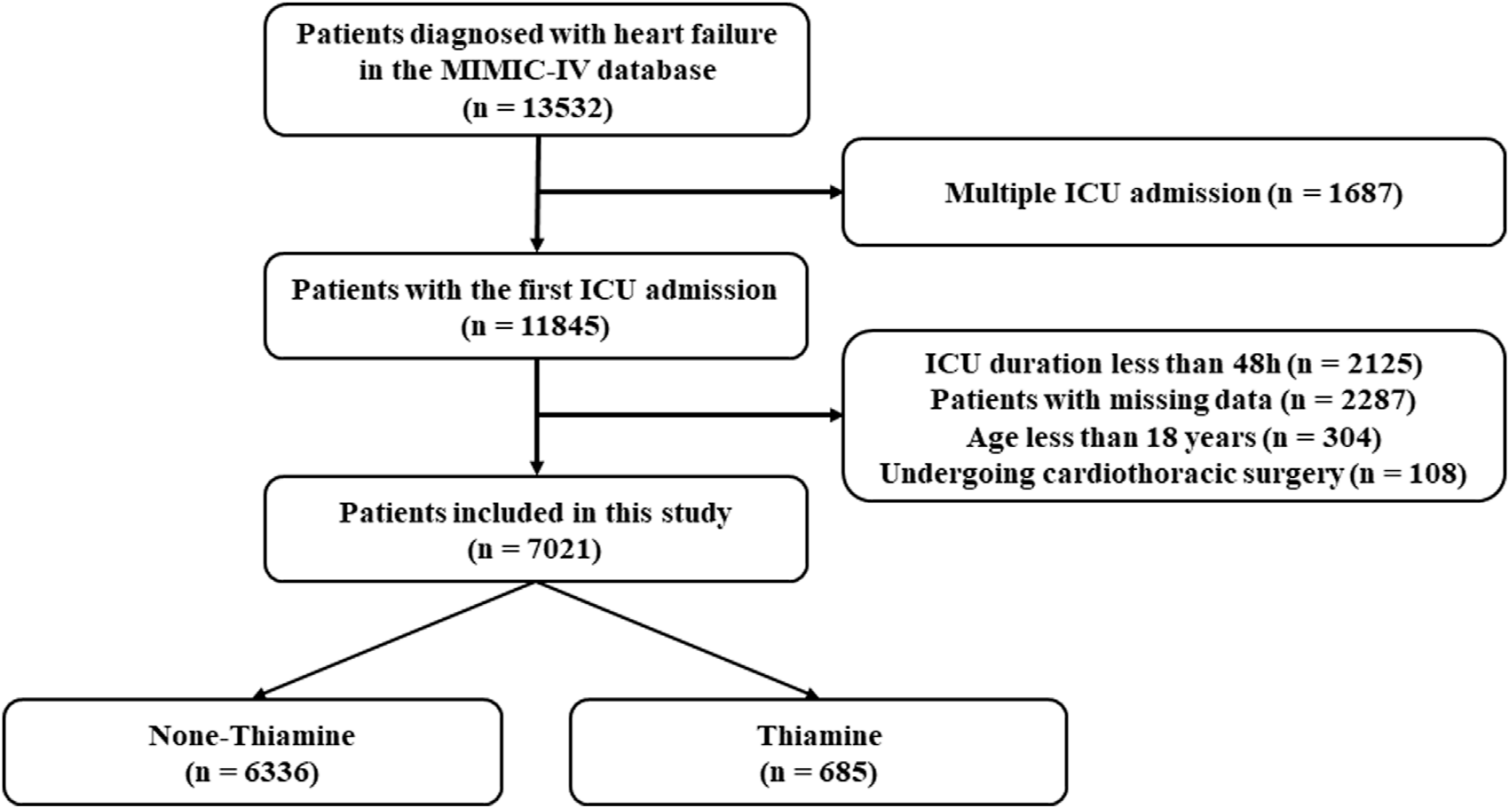
Inclusion and exclusion flowchart of the study.

As shown in Figure 1, data of 13,532 critically ill patients with heart failure were initially extracted from the MIMIC-Ⅳ database. After exclusion according to the exclusion criteria, a total of 7,021 patients were finally included in this study, consisting of 685 (9.8%) patients who received thiamine therapy during their stay in the ICUs. The median age of the original population was 76 years (IQR: 65–84) among whom 3,768 (53.7%) were male and 5,019 (71.5%) were white people.

Differences in baseline characteristics between the thiamine and non-thiamine groups are listed in Table 1. Most of the patients in the thiamine group were men, and they tended to be younger. In addition, they had a higher incidence of liver disease and sepsis but a lower rate of diabetes, chronic renal disease, and peripheral vascular disease. They also had higher values of lactate and NT-proBNP and lower values of platelets, bicarbonate, BUN, calcium, creatinine, and sodium compared with those in the non-thiamine group. Moreover, the patients in the thiamine group likely received diuretics and RRT, whereas those in the non-thiamine group were more likely to be treated with ACEIs and vasopressor.

**TABLE 1:** Baseline features of the original population.

Variable	Total (n = 7,021)	Non-thiamine (n = 6,336)	Thiamine (n = 685)	*p*-Value
Age, years	76 (65, 84)	76 (66, 85)	68 (59, 78)	<0.001
Gender, n (%)				<0.001
Male	3,768 (53.7)	3,335 (52.6)	430 (63.2)	
Female	3,253 (46.3)	3,001 (47.4)	252 (36.8)	
BMI, kg/m^2^	27.9 (24.1, 32.9)	27.8 (24.1, 32.9)	27.8 (24.4, 32.9)	0.894
Ethnicity, n (%)				<0.001
White people	5,019 (71.5)	4,574 (72.2)	445 (65.0)	
Black people	812 (11.6)	734 (11.6)	78 (11.4)	
Others	1,190 (16.9)	102 (16.2)	162 (23.6)	
Comorbidities, n (%)				
Myocardial infarct	2,103 (30.0)	1922 (30.3)	181 (26.4)	0.038
Hypertension	5,090 (72.5)	4,590 (72.4)	500 (73.0)	0.760
Diabetes	3,017 (43.0)	2,780 (42.9)	237 (34.6)	<0.001
Liver disease	744 (10.6)	570 (9.0)	174 (25.4)	<0.001
Chronic renal disease	2,944 (41.9)	2,720 (42.9)	224 (32.7)	<0.001
Peripheral vascular disease	1,253 (17.8)	1,155 (18.2)	98 (14.3)	0.013
Cerebrovascular disease	937 (13.3)	847 (13.4)	90 (13.2)	0.914
Chronic pulmonary disease	3,078 (43.8)	2,787 (44.0)	291 (42.5)	0.475
Malignant cancer	738 (10.5)	659 (10.4)	79 (11.5)	0.394
Sepsis	4,565 (65.0)	4,052 (64.0)	513 (74.0)	<0.001
Clinical scores				
Charlson comorbidity index	7 (6, 9)	8 (6, 9)	7 (5, 9)	<0.001
GCS	14 (10, 15)	14 (11, 15)	13 (9, 14)	<0.001
SOFA	5 (3, 8)	5 (3, 8)	6 (4, 9)	<0.001
APSⅢ	51 (40, 67)	51 (40, 67)	56 (43, 77)	<0.001
Vital sign				
Heart rate, beats/min	83 (73, 95)	83 (73, 94)	86 (75, 99)	<0.001
Respiratory rate, beats/min	19 (17, 22)	19 (17, 22)	20 (17, 20)	0.645
SBP, mmHg	112 (103, 125)	112 (103, 125)	111 (103, 124)	0.107
DBP, mmHg	58 (52, 65)	58 (52, 65)	60 (54, 68)	<0.001
Temperature, °C	36.7 (36.4, 37.0)	36.7 (36.4, 37.0)	36.8 (36.5, 37.1)	0.024
PaO_2_, mmHg	131.5 (86.5, 226.0)	131.5 (86.5, 226.0)	131.5 (87.5, 233.0)	0.989
PaCO_2_, mmHg	41.5 (36.5, 48.5)	41.5 (36.5, 48.5)	41.0 (35.5, 47.0)	0.297
SpO_2_, %	97 (96, 98)	97 (95, 98)	97 (96, 99)	0.038
Urine output, ml	1,470 (852, 2,345)	1,480 (855, 2,345)	1,405 (820, 2,348)	0.301
Laboratory test				
Hematocrit, %	31.5 (27.9, 35.9)	31.4 (27.0, 35.9)	31.7 (27.8, 36.2)	0.599
Hemoglobin, g/dL	10.3 (9.1, 11.8)	10.3 (9.1, 11.8)	10.4 (9.1, 12.0)	0.204
Platelets, 10^9^/L	200.5 (147.5, 266.5)	202.0 (148.5, 267.0)	190.0 (131.8, 256.0)	0.009
WBC, 10^9^/L	10.7 (7.9, 14.5)	10.7 (7.9, 14.5)	10.9 (7.5, 14.6)	0.195
Anion gap, mEq/L	14.5 (12.5, 17.0)	14.5 (12.5, 17.0)	15.0 (13.0, 17.5)	0.053
Bicarbonate, mmol/L	24.0 (21.5, 27.0)	24.5 (21.5, 27.5)	23.0 (20.0, 26.5)	<0.001
BUN, mg/dL	29.0 (19.5, 47.0)	29.5 (19.5, 47.0)	26.5 (17.0, 45.5)	0.006
Calcium, mmol/L	8.4 (7.9, 8.9)	8.4 (8.0, 8.9)	8.3 (7.8, 8.8)	<0.001
Chloride, mmol/L	103 (99.0, 107.0)	103.0 (99.0, 107.0)	103.5 (99.0, 108.0)	0.191
Creatinine, mg/dL	1.3 (1.0, 2.2)	1.4 (1.0, 2.2)	1.3 (0.9, 2.2)	0.022
Glucose, mg/dL	132.0 (110.0, 168.0)	132.5 (110.0, 168.0)	129.5 (106.5, 169.3)	0.180
Sodium, mmol/L	138.5 (135.5, 141.0)	138.5 (135.5, 141.0)	138.0 (135.5, 141.0)	0.035
Potassium, mmol/L	4.3 (3.9, 4.7)	4.3 (3.9, 4.7)	4.3 (3.9, 4.7)	0.818
pH	7.38 (7.33, 7.43)	7.38 (7.34, 7.43)	7.38 (7.32, 7.43)	0.434
Lactate, mmol/L	1.6 (1.1, 2.3)	1.6 (1.2, 2.3)	1.8 (1.3, 2.5)	<0.001
PT, seconds	14.9 (13.0, 19.1)	14.9 (13.0, 19.1)	15.1 (13.2, 18.7)	0.112
NT-proBNP, pg/mL	6,903 (2,825, 15,550)	6,169 (2,583, 14,007)	7,450 (3,075, 16,441)	0.004
Clinical Therapy, n (%)				
ACEIs	2,199 (31.3)	2021 (31.9)	178 (26.0)	0.002
ARBs	416 (5.9)	385 (6.1)	31 (4.5)	0.102
ICD	138 (2.0)	122 (1.9)	16 (2.3)	0.462
Beta-blockers	2,531 (36.1)	2,281 (36.0)	250 (36.5)	0.797
Diuretics	5,378 (76.6)	4,824 (76.1)	554 (80.9)	0.005
Vasopressor	6,704 (95.5)	6,065 (95.7)	639 (93.3)	0.005
RRT	718 (10.2)	621 (9.8)	97 (14.2)	<0.001
Mechanical ventilation	3,639 (51.8)	3,290 (51.9)	349 (50.9)	0.627
ICU length of stay, day	3.9 (2.1, 9.0)	3.4 (1.9, 7.9)	5.2 (2.5, 10.3)	<0.001
Hospital length of stay, day	10.5 (5.9, 17.2)	9.8 (5.5, 16.5)	12.1 (6.4, 18.7)	<0.001

ACEIs, angiotensin converting enzyme inhibitors; ARBs, angiotensin receptor blockers; APS, acute physiology score; BMI, body mass index; BUN, blood urea nitrogen; DBP, diastolic blood pressure; GCS, glasgow coma scale; ICD, implantable cardioverter defibrillator; ICU, intensive care unit; NT-proBNP, N-terminal pro-brain natriuretic peptide; PaCO_2_, partial pressure of carbon dioxide; PaO_2_, partial pressure of oxygen; pH, hydrogen ion concentration; PT, prothrombin time; RRT, renal replacement therapy; SBP, systolic blood pressure; SOFA, sequential organ failure assessment; SpO2, oxygen saturation; WBC, white blood cell.

After PSM, a total of 681 patients who received thiamine therapy were matched to 681 cases who didn’t, and no difference in baseline characteristics was found between the two groups (Supplementary Table S1). A scatter plot based on the propensity score showed the matched and the unmatched cases in the two groups, indicating a good quality of the matched samples (Supplementary Figure S1A). Moreover, the histogram used to show the distribution of propensity score also suggested that the basic shapes of the two groups are highly consistent after matching, which further validated the matching effect (Supplementary Figure S1B).

### Survival analysis

**FIGURE 2:**
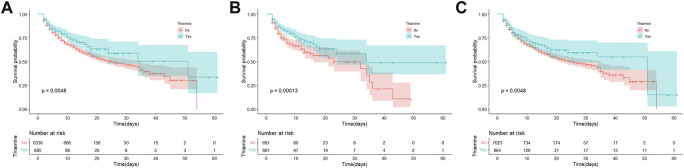
Kaplan-Meier survival curves between the thiamine and none-thiamine groups. **(A)** The original population; **(B)** After propensity score matching adjustment; and **(C)** After propensity score-based inverse probability of treatment weighting adjustment.

A total of 100 (14.6%) and 1,166 (18.4%) patients died during hospitalization in the thiamine and none-thiamine groups, respectively. As shown in Figure 2A, the thiamine group had a lower in-hospital mortality than the non-thiamine group in the original population (*p* = 0.005). After PSM (*p* < 0.001; Figure 2B) and IPW (*p* = 0.005; Figure 2C), the results of KM survival curves were consistent with that of the original population.

Considering that all VIFs were less than 5, no multicollinearity was determined among variables (Supplementary Table S3). We further analyzed the relationship between thiamine supplement and prognosis through Cox proportional hazard models and the results are listed in Table 2. A crude model of univariate Cox regression analysis revealed that thiamine use was significantly associated with a 26% reduction in the risk of in-hospital mortality in the original population with a HR of 0.74 (95% CI: 0.64–0.84, *p* < 0.001). After adjusting for a series of confounders, multivariate analyses indicated a significant beneficial effect of thiamine administration on in-hospital mortality among critically ill patients with heart failure with a HR of 0.78 (95% CI: 0.67–0.89, *p* = 0.004) in the fully adjusted model. After PSM and IPW, the crude models demonstrated that thiamine use was related to a decreased mortality risk with HRs (95% CIs) of 0.61 (95% CI: 0.48–0.79, *p* < 0.001) and 0.79 (95% CI: 0.68–0.90, *p* = 0.004), respectively. The PSM and IPW models also showed similar results with HRs of 0.68 (95% CI: 0.56–0.89, *p* < 0.001) and 0.84 (95% CI: 0.73–0.96, *p* = 0.035) in the fully adjusted models, respectively.

**TABLE 2:** Results of Cox proportional hazard models.

Models	Original population		PSM population		IPW population	
	HR (95% CI)	*p*-Value	HR (95% CI)	*p*-Value	HR (95% CI)	*p*-Value
Crude model	0.74 (0.64–0.84)	<0.001	0.61 (0.48–0.79)	<0.001	0.79 (0.68–0.90)	0.004
Model 1	0.74 (0.64–0.84)	<0.001	0.61 (0.48–0.79)	<0.001	0.79 (0.69–0.90)	0.004
Model 2	0.76 (0.65–0.87)	<0.001	0.64 (0.50–0.83)	<0.001	0.80 (0.69–0.92)	0.011
Model 3	0.75 (0.64–0.87)	<0.001	0.65 (0.51–0.84)	<0.001	0.82 (0.70–0.94)	0.020
Model 4	0.76 (0.65–0.88)	0.001	0.64 (0.51–0.84)	<0.001	0.82 (0.70–0.94)	0.021
Model 5	0.77 (0.66–0.89)	0.002	0.67 (0.55–0.88)	<0.001	0.84 (0.72–0.96)	0.030
Model 6	0.78 (0.67–0.89)	0.004	0.68 (0.56–0.89)	<0.001	0.84 (0.73–0.96)	0.035

HR, hazard ratio; CI, confidence interval; PSM, propensity-score matching; IPW, inverse probability weighting.

^a^ Model 1 was adjusted for demographic features, including age, gender, ethnicity, and BMI.

^b^ Model 2 was additionally adjusted for comorbidities, including myocardial infarct, hypertension, diabetes, liver disease, chronic renal disease, peripheral vascular disease, cerebrovascular disease, chronic pulmonary disease, malignant cancer, and sepsis.

^c^ Model 3 was additionally adjusted for clinical scores, including Charlson comorbidity index, GCS, SOFA, and APSⅢ.

^d^ Model 4 was additionally adjusted for vital signs, including heart rate, respiratory rate, SBP, DBP, temperature, PO_2_, PCO_2_, SpO_2_, and urine output.

^e^ Model 5 was additionally adjusted for laboratory tests, including hematocrit, hemoglobin, platelets, white blood cell, anion gap, bicarbonate, BUN, calcium, chloride, creatinine, glucose, sodium, potassium; PH, lactate; PT, and NT-proBNP.

^f^ Model 6 was additionally adjusted for clinical therapy, including ACEI, ARB, ICD, beta-blocker, diuretics, renal replacement therapy, mechanical ventilation, and vasopressor.

### Subgroup analysis

**FIGURE 3:**
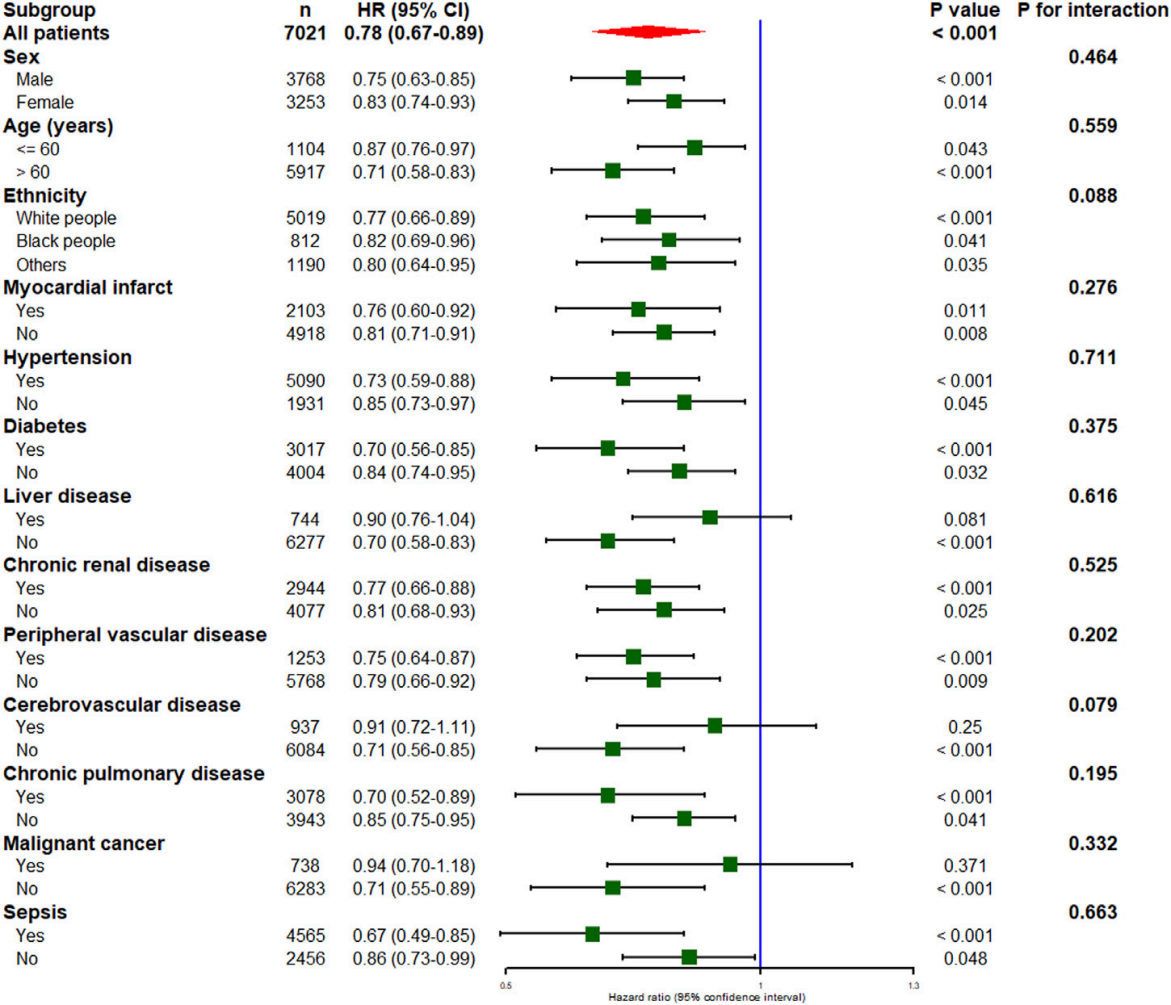
Subgroup analysis of the association between thiamine use and in-hospital mortality in critically ill patients with heart failure. HR, hazard ratio; CI, confidence interval.

The results of subgroup analyses are listed in Figure 3. A significant association between thiamine use and in-hospital mortality was observed in all subgroups except for patients with liver disease, cerebrovascular disease, or malignant cancer. In addition, no significant interaction was observed between the thiamine and none-thiamine groups in all strata.

## Discussion

Hospital admissions and mortality caused by heart failure are high. The treatment of patients with critical heart failure is expensive, and although several resources have been devoted to the development of drugs for the treatment of critical heart failure worldwide, the prognosis is still not ideal. Clinical recommendations primarily focus on limiting fluid and salt, as well as energy and protein intake, whereas recommendations for the supplementation of micronutrients, such as mineral and vitamin, are limited (van der Meer et al., 2019). Nutritional supplementation is economical and safe and, if proven effective, can significantly reduce the burden of heart failure. Thiamine supplementation can improve the state of heart failure caused by severe thiamine deficiency, but whether thiamine supplementation is effective in critically ill patients with heart failure remains unknown (Costa et al., 2022). Therefore, this study aimed to explore the correlation between thiamine use and prognosis in patients with HF. After controlling for potential confounding factors using Cox regression models, we found that thiamine supplementation in critically ill patients with heart failure could significantly decrease the risk of in-hospital mortality. The results of dual robustness tests for PSM and IPW also supported this finding.

Critically ill patients who were admitted to the ICUs are often in a state of fasting or undereating because of their critical condition, leading to inadequate thiamine intake. In particular, patients with obesity who routinely take multivitamin supplements aren’t immune to thiamine deficiency (Manzanares and Hardy, 2011). In this study, critically ill patients with heart failure who were admitted to the ICUs were all over 55 years old, and overweight. Most of them had multiple complications, indicating that they had a high risk of thiamine deficiency. However, only less than 10% of these patients had received thiamine supplement, which was significantly lower than those in patients with sepsis and ventilator-associated pneumonia (Hu et al., 2022; Zhang et al., 2022). This apparent difference may be related to the variability of care providers. Considering that heart failure is a fatal disease, healthcare professionals tend to focus more on heart failure treatment. Moreover, although some guidelines and consensus suggest thiamine supplementation in patients with heart failure, recommendations regarding specific reference intake and ingestion methods vary, and clinical trials have not yielded consistent results. This may explain the lower-than-expected thiamine supplementation rate in patients with heart failure.

The human body cannot synthesize thiamine itself, and it has limited stocks; thus, it must rely on external sources for replenishment. Thiamine, as a key coenzyme in glycolysis, plays a key regulatory role in mitochondrial ATP synthesis and provides energy for cells; thus, the lack of thiamine could affect mitochondrial function (Page et al., 2011). Impaired mitochondrial function can cause cellular dysfunction, leading to serious complications of heart failure, neuropathy, gastrointestinal dysfunction, and lactic acidosis (Zhou and Tian, 2018; Eftekharpour and Fernyhough, 2022). Therefore, thiamine supplementation helps restore mitochondrial function and perfusion of damaged tissues, thereby reducing the likelihood of organ dysfunction and improving patient prognosis. Apart from its vital role in energy metabolism, thiamine is also an antioxidant. In critically ill patients, changes in cell structure and the imbalance of oxidative and antioxidant systems lead to high levels of oxidative stress products such as reactive oxygen species, because of inflammatory response and tissue hypoxia (Zanza et al., 2019). Thiamine supplementation can reduce oxidative stress by inhibiting lipid peroxidation and oleic acid oxidation, thereby positively affecting oxidative stress level (Collie et al., 2017). Thiamine also has an antioxidant effect on neutrophil cells and a protective effect on macrophages against oxidative stress-induced NF-kB activation, and it plays an important role in the activity of p53 suppressor protein by inhibiting the intracellular activity of P43, thereby exerting an anti-inflammatory effect (Mitchell et al., 2020).

In this study, a close association was found between thiamine administration and a decreased risk of in-hospital mortality in critically ill patients with heart failure, regardless of adjustments on various confounders. After multiple robust verifications and subgroup analyses, the results all confirmed the important significance of thiamine supplementation in improving the prognosis among these patients. Moreover, based on the comparison of baseline characteristics between the thiamine and non-thiamine groups, thiamine supplement can reduce the use of ventilators, ACEI, and vasopressors in critically ill patients with heart failure, indicating that it is a safe and effective treatment.

This study has some advantages. First, electronic medical records from the MIMIC-IV database were used, which have a high focus on patients admitted to ICUs with a large sample size, thereby providing strong evidence for our conclusions. Second, we obtained the same result after adjusting the baseline level by using PSM and IPW and establishing a series Cox regression models to adjust various confounding factors, which further confirmed the reliability of the results (Zhong et al., 2022). However, this study also has some limitations. First, this study had a single-center retrospective observational design; thus, selection bias was inevitable (Yue et al., 2022). Second, we cannot identify thiamine deficiency because of the lack of baseline thiamine levels in MIMIC-Ⅳ. Therefore, we cannot infer whether all patients with heart failure benefit, or only individuals with thiamine deficiency. Third, we only grouped patients based on whether they received in-hospital thiamine supplementation or not, and we didn’t consider dosage and duration of administration, which may limit the application of our findings. Fourth, the adjustments in our study may not be sufficient to address all confounding variables, and some confounding factors may remain unexplained, such as the variability of care providers. Despite a significant difference in mortality, the present results should be interpreted with caution. Finally, considering that the MIMIC-Ⅳ database rarely records lactate levels in patients, we cannot determine whether thiamine improved patient outcomes by reducing lactate concentrations.

## Conclusion

Thiamine supplementation is beneficial to the prognosis of critically ill patients with heart failure who were admitted in ICUs. Given its low cost and relatively few side effects, thiamine supplementation may be useful in these patients. However, the results are not conclusive, and they should be validated by further clinical trials with large sample sizes.

## Data Availability

The original contributions presented in the study are included in the article/Supplementary Material, further inquiries can be directed to the corresponding authors.

## Supplementary material

SUPPLEMENTARY FIGURE S1 Baseline difference between the thiamine and non-thiamine groups before and after propensity score matching.
