# Supplementary material for: Association of thiamine administration and prognosis in critically ill patients with heart failure
Source: Front Pharmacol. 2023 Mar 23;14:1162797. doi: 10.3389/fphar.2023.1162797 (PMC10076601; doi:10.3389/fphar.2023.1162797)
Supplement: Supplementary file 2 [file Table1.DOCX]

**Supplementary Table 1. Baseline features of the PSM population.**

| **Variable** | **Total**  **(n = 1362)** | **None-Thiamine**  **(n = 681)** | **Thiamine**  **(n = 681)** | ***P* value** |
| --- | --- | --- | --- | --- |
| Age, years | 68 (58, 79) | 68 (57, 80) | 68 (59, 78) | 0.786 |
| Gender, n (%) |  |  |  | 0.576 |
| Male | 848 (62.3) | 3335 (52.6) | 430 (63.2) |  |
| Female | 514 (37.7) | 3001 (47.4) | 252 (36.8) |  |
| BMI, kg/m^2^ | 27.8 (24.1, 32.9) | 27.7 (23.6, 32.9) | 27.8 (24.4, 32.9) | 0.745 |
| Ethnicity, n (%) |  |  |  | 0.844 |
| White | 896 (65.8) | 4574 (72.2) | 445 (65.0) |  |
| Black | 157 (11.5) | 734 (11.6) | 78 (11.4) |  |
| Others | 309 (22.7) | 102 (16.2) | 162 (23.6) |  |
| Comorbidities, n (%) |  |  |  |  |
| Myocardial infarct | 356 (26.1) | 1922 (30.3) | 181 (26.4) | 0.805 |
| Hypertension | 993 (72.9) | 495 (72.9) | 498 (73.1) | 0.855 |
| Diabetes | 458 (33.6) | 2780 (42.9) | 237 (34.6) | 0.359 |
| Liver disease | 358 (26.3) | 570 (9.0) | 174 (25.4) | 0.268 |
| Chronic renal disease | 464 (34.1) | 2720 (42.9) | 224 (32.7) | 0.360 |
| Peripheral vascular disease | 205 (15.1) | 1155 (18.2) | 98 (14.3) | 0.495 |
| Cerebrovascular disease | 181 (13.3) | 847 (13.4) | 90 (13.2) | 0.936 |
| Chronic pulmonary disease | 597 (43.8) | 2787 (44.0) | 291 (42.5) | 0.299 |
| Malignant cancer | 168 (12.3) | 659 (10.4) | 79 (11.5) | 0.410 |
| Sepsis | 1004 (73.7) | 4052 (64.0) | 513 (74.0) | 0.389 |
| Clinical scores |  |  |  |  |
| Charlson comorbidity index | 7 (5, 9) | 7 (5, 9) | 7 (5, 9) | 0.442 |
| GCS | 13 (9, 14) | 14 (9, 15) | 13 (9, 14) | 0.060 |
| SOFA | 6 (4, 9) | 6 (4, 9) | 6 (4, 9) | 0.914 |
| APSⅢ | 55 (41, 77) | 54 (40, 79) | 56 (42, 76) | 0.357 |
| Vital sign |  |  |  |  |
| Heart rate, beats/min | 86 (75, 98) | 86 (76, 98) | 86 (75, 99) | 0.957 |
| Respiratory rate, beats/min | 19 (17, 23) | 19 (17, 23) | 19 (17, 23) | 0.828 |
| SBP, mmHg | 111 (103, 124) | 111 (103, 124) | 111 (103, 124) | 0.626 |
| DBP, mmHg | 60 (54, 68) | 60 (54, 69) | 60 (54, 68) | 0.588 |
| Temperature, ℃ | 36.8 (36.4, 37.1) | 36.7 (36.4, 37.1) | 36.8 (36.5, 37.1) | 0.924 |
| PaO_2_, mmHg | 136.5 (89.0, 226.0) | 141.0 (90.0, 224.5) | 131.5 (87.5, 228.0) | 0.193 |
| PaCO_2_, mmHg | 41.0 (35.5, 47.0) | 41.0 (36.0, 47.0) | 41.0 (35.5, 47.0) | 0.828 |
| SpO_2_, % | 97 (96, 99) | 97 (95, 99) | 97 (96, 99) | 0.827 |
| Urine output, ml | 1475 (833, 2403) | 1490 (840, 2345) | 1405 (823, 2340) | 0.143 |
| Laboratory test |  |  |  |  |
| Hematocrit, % | 31.6 (27.8, 36.2) | 31.6 (27.8, 36.2) | 31.6 (27.8, 36.2) | 0.871 |
| Hemoglobin, g/dl | 10.4 (9.1, 11.9) | 10.4 (9.1, 11.9) | 10.4 (9.0, 12.0) | 0.957 |
| Platelets, 10^9^/L | 192.0 (136.9, 255.1) | 192.0 (140.5, 251.5) | 191.5 (132.0, 256.5) | 0. 914 |
| WBC, 10^9^/L | 10.8 (7.8, 14.6) | 10.6 (8.0, 14.5) | 11.0 (7.5, 14.6) | 0.329 |
| Anion gap, mEq/L | 15.0 (13.0, 17.5) | 15.0 (13.0, 17.5) | 15.0 (13.0, 17.5) | 0.663 |
| Bicarbonate, mmol/L | 23.0 (20.5, 26.5) | 23.5 (20.5, 26.5) | 23.0 (20.5, 26.5) | 0.357 |
| BUN, mg/dl | 27.5 (18.0, 45.5) | 28.0 (19.0, 46.0) | 26.5 (17.0, 45.0) | 0.212 |
| Calcium, mmol/L | 8.3 (7.8, 8.8) | 8.4 (7.8, 8.8) | 8.3 (7.8, 8.8) | 0.193 |
| Chloride, mmol/L | 103.5 (99.0, 107.5) | 104.0 (99.0, 107.0) | 103.5 (99.0, 108.0) | 0.828 |
| Creatinine, mg/dl | 1.3 (0.9, 2.2) | 1.3 (0.9, 2.2) | 1.3 (0.9, 2.2) | 0.302 |
| Glucose, mg/dl | 130.0 (108.8, 163.6) | 130.0 (110.0, 160.0) | 130.0 (106.5, 169.8) | 0.957 |
| Sodium, mmol/L | 138.0 (135.5, 141.0) | 138.0 (135.0, 141.0) | 138.0 (135.5, 141.0) | 0.481 |
| Potassium, mmol/L | 4.2 (3.9, 4.7) | 4.2 (3.9, 4.6) | 4.3 (3.9, 4.7) | 0.386 |
| pH | 7.38 (7.33, 7.43) | 7.38 (7.33, 7.43) | 7.38 (7.33, 7.43) | 0.994 |
| Lactate, mmol/L | 1.8 (1.3, 2.6) | 1.8 (1.2, 2.7) | 1.8 (1.3, 2.5) | 0.551 |
| PT, seconds | 15.2 (13.3, 19.2) | 15.2 (13.4, 19.5) | 15.1 (13.2, 18.7) | 0.386 |
| NT-proBNP, pg/ml | 7203 (2981, 15712) | 7055 (2899, 15517) | 7238 (3022, 16110) | 0.225 |
| Clinical Therapy, n (%) |  |  |  |  |
| ACEI | 368 (27.0) | 190 (27.9) | 178 (26.1) | 0.464 |
| ARB | 66 (4.8) | 35 (5.1) | 31 (4.6) | 0.606 |
| ICD | 29 (2.1) | 14 (2.1) | 15 (2.2) | 0.851 |
| Beta-blocker | 494 (36.3) | 246 (36.1) | 248 (36.4) | 0.910 |
| Diuretics | 1094 (80.3) | 544 (79.9) | 550 (80.8) | 0.683 |
| Vasopressor | 1278 (93.8) | 641 (94.1) | 637 (93.5) | 0.652 |
| RRT | 192 (14.1) | 99 (14.5) | 93 (13.7) | 0.640 |
| Mechanical ventilation | 695 (51.0) | 350 (51.4) | 345 (50.7) | 0.448 |

ACEI, angiotensin, converting enzyme inhibitor; ARB, angiotensin receptor blocker; APS, acute physiology score; BMI, body mass index; BUN, blood urea nitrogen; DBP, diastolic blood pressure; GCS, Glasgow Coma Scale; ICD, implantable cardioverter defibrillator; NT-proBNP, N-terminal pro-brain natriuretic peptide; PaCO_2_, partial pressure of carbon dioxide; PaO_2_, partial pressure of oxygen; pH, hydrogen ion concentration; PT, prothrombin time; RRT, renal replacement therapy; SBP, systolic blood pressure; SOFA, sequential organ failure assessment; SpO2, oxygen saturation; WBC, white blood cell.
