# Supplementary figures and images for: Association of thiamine administration and prognosis in critically ill patients with heart failure
Source: Front Pharmacol. 2023 Mar 23;14:1162797. doi: 10.3389/fphar.2023.1162797 (PMC10076601; doi:10.3389/fphar.2023.1162797)

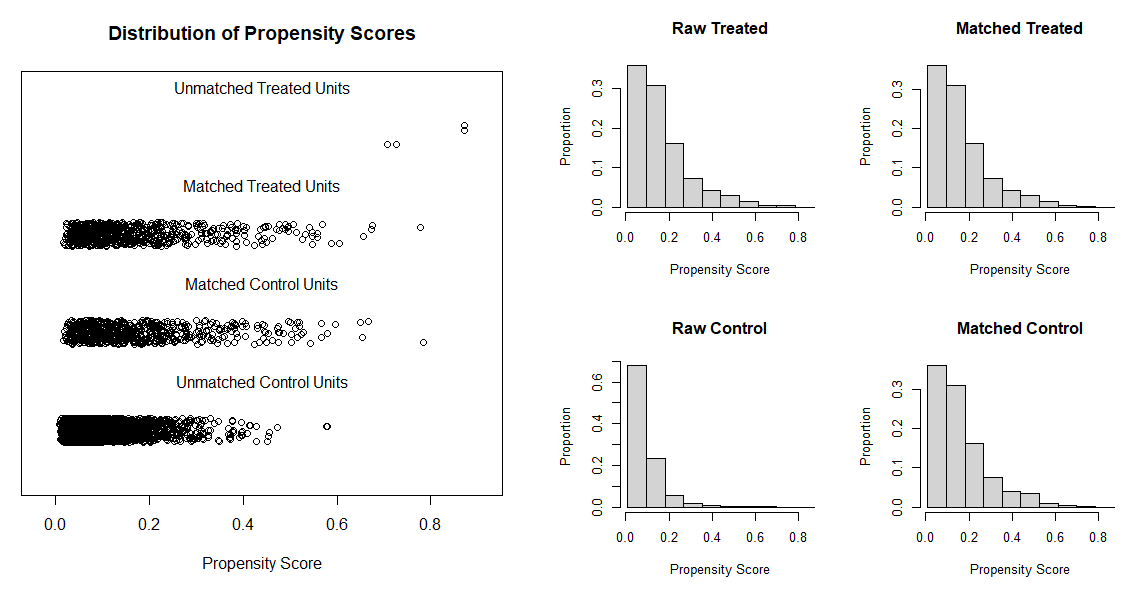

Supplement: Supplementary file 3 [file Image1.TIF]
